# The impacts of land cover types on urban outdoor thermal environment: the case of Beijing, China

**DOI:** 10.1186/s40201-015-0195-x

**Published:** 2015-05-14

**Authors:** Hai Yan, Li Dong

**Affiliations:** College of Landscape Architecture, Beijing Forestry University, Beijing, 100083 China; School of Landscape Architecture, Zhejiang Agriculture and Forestry University, Lin’an, Zhejiang 311300 China

**Keywords:** Urban heat island, Urban park, Vegetation, Air temperature, Land cover

## Abstract

**Background:**

This study investigated the microclimatic behavior of different land cover types in urban parks and, the correlation between air temperature and land cover composition to understand how land cover affects outdoor thermal environment during hot summer.

**Methods:**

To address this issue, air temperatures were measured on four different land cover types at four observation sites inside an urban park in Beijing, China, meanwhile, the land cover composition of each site was quantified with CAD, by drawing corresponding areas on the aerial photographs.

**Results:**

The results showed that the average air temperature difference among four land cover types was large during the day and small during the night. At noon, the average air temperature differed significantly among four land cover types, whereas on night, there was no significant difference among different land cover types. Results of the linear regression indicated that during daytime, there was a strong negative correlation between air temperature and percent tree cover; while at nighttime, a significant negative correlation was observed between air temperature and percent lawn cover. It was shown that as the percent tree cover increased by 10 %, the air temperature decreased by 0.26 °C during daytime, while as the percent lawn cover increased by 10 %, the air temperature decreased by 0.56 °C during nighttime.

**Conclusions:**

Results of this study help to clarify the effects of land cover on urban outdoor thermal environment, and can provide assistance to urban planner and designer for improving green space planning and design in the future.

## Introduction

In the last decades, a great concentration of people around urban areas took place worldwide. The urbanization process, with its fast population increase, creates changes in the urban climate [1]. A distinct feature of urban climate is the urban heat island (UHI) effect, in which the urban air temperature is higher than the air temperature of the surrounding rural or suburban areas [2]. UHI has significant negative effects on the buildings energy consumption, outdoor air quality, living environment, and habitability of cities [3, 4]. With increasing urbanization, the urban heat island will affect a larger number of urban residents. Therefore, there is a pressing need for urban researchers to evaluate strategies that may mitigate against further increases in temperatures in urban areas.

Among all cooling measures, planting of vegetation in urban areas is one of the simplest and most effective strategies to mitigate the UHI effect [5, 6]. Urban green areas can ameliorate UHI effect by the combined impact of shading and evapotranspiration. A series of field measurement studies show that vegetated areas are likely to be cooler than their surrounding urban built environment [7–10]. This vegetated cool patch within the warmer built-up environment is referred to as a “park cool island” (PCI). In a study, by Jansson et al. [11], temperatures of an urban vegetated park and its surroundings in central Stockholm were measured during three summer days, the results indicated that the PCI intensity was in the range of 0.5–0.8 °C during the day and reached a maximum of 2 °C at sunset. In Mexico City, Jauregui [12] found that the air temperature of a large irrigated park was 3–4 °C cooler than its built-up surroundings. Similar results were also obtained from a study of two urban parks in Singapore [13]. The cooling effect of urban parks in cities has been confirmed by the above studies. However, the physical constitution of the parks, such as the type of pavements, the amount of tree and grass cover and the floristic structure of vegetation can be expected to affect the cooling effect [14]. Cao et al. [15], based on remote sensing data, studied the effects of park characteristics on the formation of PCI in Nagoya, and found that PCI intensity is clearly related to the area of tree and shrub inside the park, and that grass has negative impact on PCI formation. Comparing the temperatures of 61 parks during the summer at noon in Taipei City, Chang et al. [16] found that parks with more than 50 % paved coverage and little tree and shrub cover were on average warmer than their surroundings. The land cover composition of a park has been shown to positively correlate with the magnitude of differences in air temperature.

From these findings, it can be inferred that park characteristics, especially in regard to the type of land cover and the proportion of different land cover types may have a significant effect on outdoor air temperature [17, 18]. Therefore, knowledge of the relationship between air temperature and land cover is critical in improving landscape design strategies to ameliorate urban thermal environment. However, the quantifiable effect and statistical relationship between air temperature and land cover in a park have not been established and thus limit the design of optimal parks with remarkable PCI effect. In the present study, we investigated the thermal performance of different land cover types, and how the feature of land cover composition influences local air temperature in the city of Beijing, China. The work focused on humidity and hot summer days, when the green areas are more frequently used by the citizens in the study area.

The objectives of this study were:

1. to examine diurnal variations in air temperature of different land cover types;
2. to compare the air temperature difference among various land cover types;
3. to quantify the relationship between air temperature and land cover composition during daytime and nighttime in summer.

## Methods

### Study area and site description

Beijing is located in the northern part of the North China Plain. It is the second largest city in China with a total population of 19.6 million by the end of 2010. It has a monsoon influenced humid continental climate characterized by hot and humid summers and generally cold, windy and dry winters. According to the climatological normals (1971–2000), January is the coldest month with an average temperature of −3.7 °C, while July is the hottest month with an average temperature of 26.2 °C. The main wind direction is from southeast to northeast in summer and in reverse during winter. Since 1978, Beijing’s urban population and yearly construction area have been gradually increasing, leading to significant modifications in the underlying surface properties and notable increase in the intensity of the UHI effect.

**Fig. 1:**
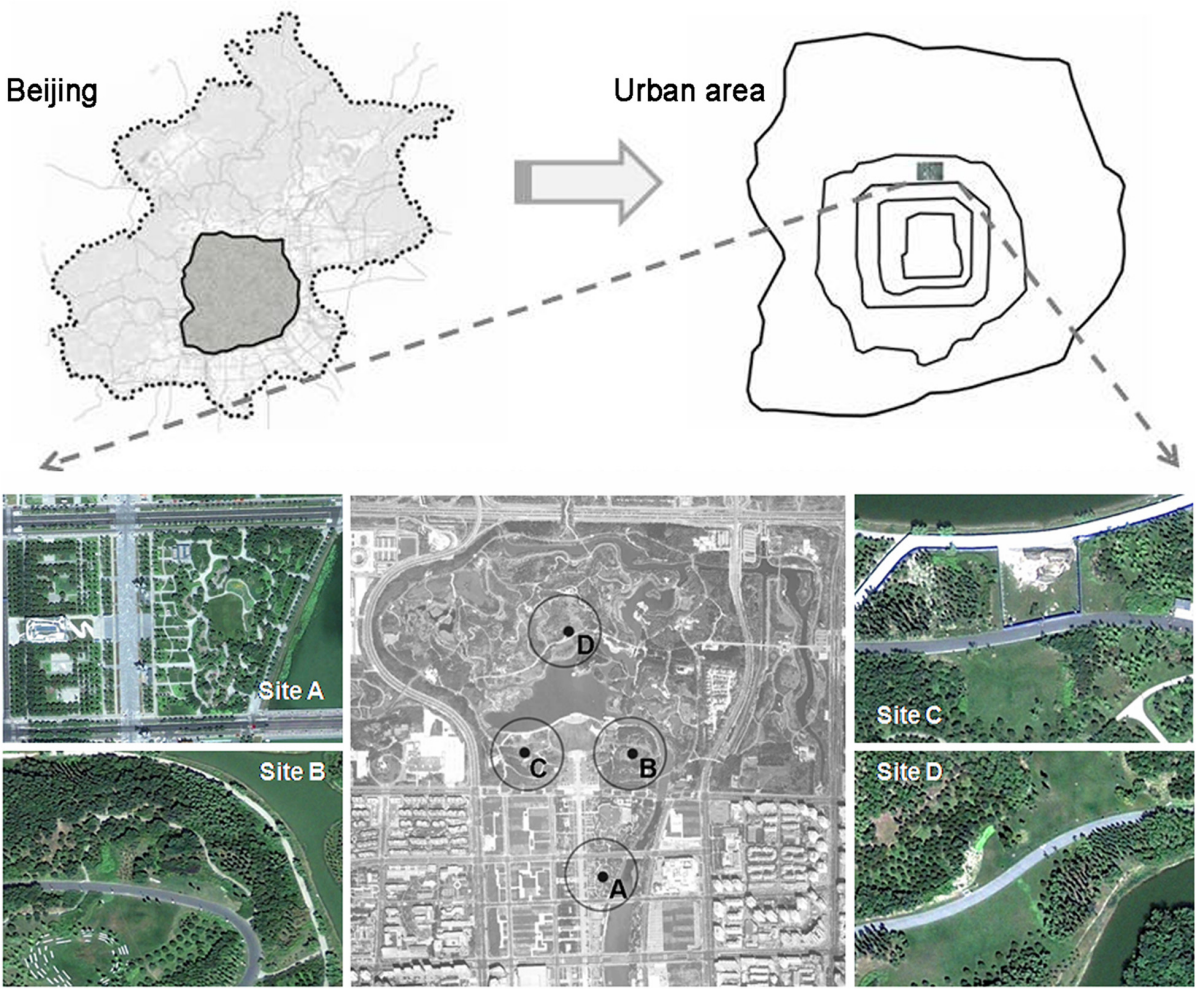
Location and aerial view of the observation sites

This study was conducted in the Beijing Olympic Forestry Park, which is in the northern part of the city and surrounded by residential and commercial buildings. The park covers 680 ha; about 70 % of the total area is vegetated area and 10 % is water area. Observations were conducted simultaneously on four fixed sites. The locations of the four observation sites are indicated in Fig. 1, and site A, one of the measurement sites, is outside the park and the other three sites (B, C and D) are inside the park. At each observation site we selected four types of land cover, i.e. trees area, lawn area, paved area and water area as sample points to conduct air temperature measurements. The method similar to Krüger et al. [19] was utilized to obtain land cover pattern of each observation site, which were made with CAD, by drawing corresponding areas on the aerial photographs. Each observation site represents an area of 49,000 m^2^ (approximately a 125 m radius around the center of each observation site). Table 1 shows the percentages of the four categories of land cover for each observation site.

**Table 1:** Land cover percentages for each observation site (%)

Location	Wood area	Lawn area	Paved area	Water area
Site A	20.1	24.9	44.7	10.3
Site B	47.0	33.5	10.4	9.1
Site C	52.6	26.9	8.1	12.4
Site D	59.4	24.1	5.6	10.9

### Air temperature measurements

Measurements were carried out simultaneously at four observation sites on 18th, 27th, 28th, July of 2010. Atmospheric conditions on the measuring days were fairly clear and windless. During the observations, air temperature was measured once every two hours from 8:00 to 20:00 h. The major instruments used in the measurement were Testo humidity temperature meters. The measurements were taken at 1.5 m above the ground, which is approximately the level where a person of mean height breathes in. At each measurement point, we waited until the wind speed slowed below 2 m/s and wait for an additional few minutes for the temperature measurement to stabilize before taking the air temperature data.

### Data analysis

In the data analysis, 14:00 h and 20:00 h were selected to represent the daytime and nighttime, respectively. Statistical analyses were performed to test the influence of land cover on air temperature using one-way ANOVA, and multiple comparisons were made using the Turkey HSD method. Simple correlation analysis was also performed to study the relations between local air temperature and land cover patterns of each observation site during daytime and nighttime. Statistical analysis was performed by running the SPSS/PC + software package (SPSS, Inc.) on a personal computer. P values of less than 0.05 were regarded as statistically significant.

## Results and discussion

### Temperature difference among land cover types

**Fig. 2:**
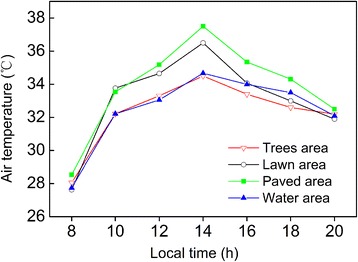
The diurnal variation of air temperature at four types of land cover during hot summer

Figure 2 shows the air temperature variation of different land cover types during hot summer. Temperature difference among land cover types was minimal in the morning, increased during the hot hours of the day and then decreased toward the evening. In general, three main temperature patterns could be observed: the paved area had the highest temperature values, the lawn area was slightly cooler than the paved area and, air temperatures were lowest in the water area and trees area. It is evident that the air temperature of all four land cover types reached the highest value at 14:00 h, and the highest temperature value is in the order of paved area > lawn area > water area > trees area. They were 37.5 °C in the paved area, 36.5 °Cin the lawn area, 34.7 °Cin the water area and 34.5 °Cin the trees area. In the late afternoon, air temperature of lawn area dropped fastest among the four land cover types, which was likely to became the same with that of trees area at about 19:00 h, and reached the minimum (36.1 °C) at 20:00 h.

**Fig. 3:**
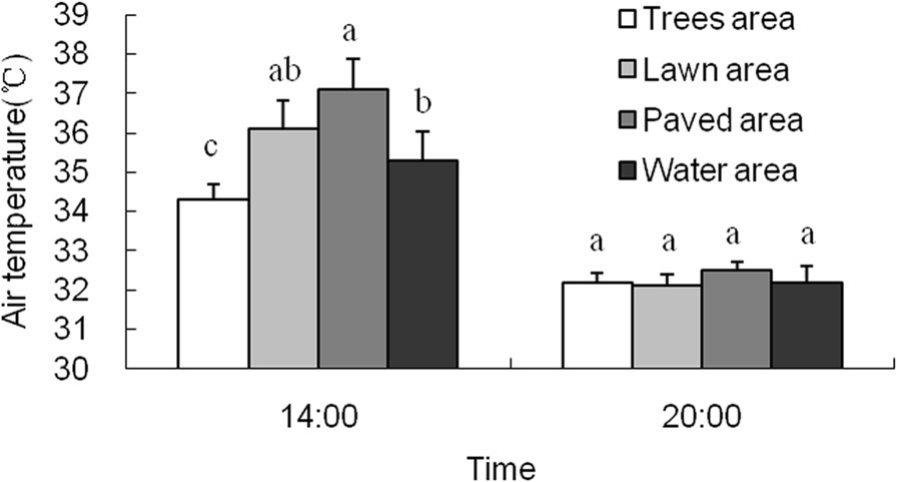
Average air temperature of four land cover types at two time periods. Lowercase letters indicate significantly different means based on a Tukey HSD multiple comparisons test at 0.05 level. Values in each column with the same letter are not significantly different among land covers

Considering the air temperature difference of each land cover type during different time periods, daytime and nighttime data were analyzed separately. In this study, temperature at 14:00 h and 20:00 were selected to represent the data of daytime and nighttime, respectively. Figure 3 illustrates the average air temperatures of each land cover type during the two time periods. At 14:00 h, difference in average air temperatures was significant among different land cover types. The average air temperature of paved area (37.1 °C) was significantly higher than that of water area (35.3 °C) and trees area (34.3 °C), meanwhile, temperature of lawn area (36.1 °C) and water area were markedly higher than that of trees area. Previous studies carried out in Nanjing by Huang et al. [20] also found similar temperature differences among different land cover types. During daytime, the effect of solar radiation was the main reason for this temperature behavior. The lower air temperature in trees area was the result of good shading provided by dense canopy, which was able to intercept much more incoming solar radiation when compared to open paved area and lawn area [21]. Both paved area and lawn area were constantly exposed to direct solar radiation, resulting in increased temperature. At 20:00 h, the paved area was still found to be the warmest one, while the lawn became the coolest site. However, there was no significant difference in average air temperatures among four land cover types at this time. It was also found that the maximum nighttime temperature difference (0.4 °C) among various land cover types is lower than that of the daytime period (2.8 °C). During daytime, the large temperature difference was mainly contributed by the high air temperature which was located at open paved area and the low temperature which was located at shaded areas with vegetation; whereas, during nighttime, the former site cool down at a faster rate as compared with the latter, since there was no obstruction to block the nighttime net long-wave loss without vegetation.

### Relationship between local air temperature and land cover pattern

**Fig. 4:**
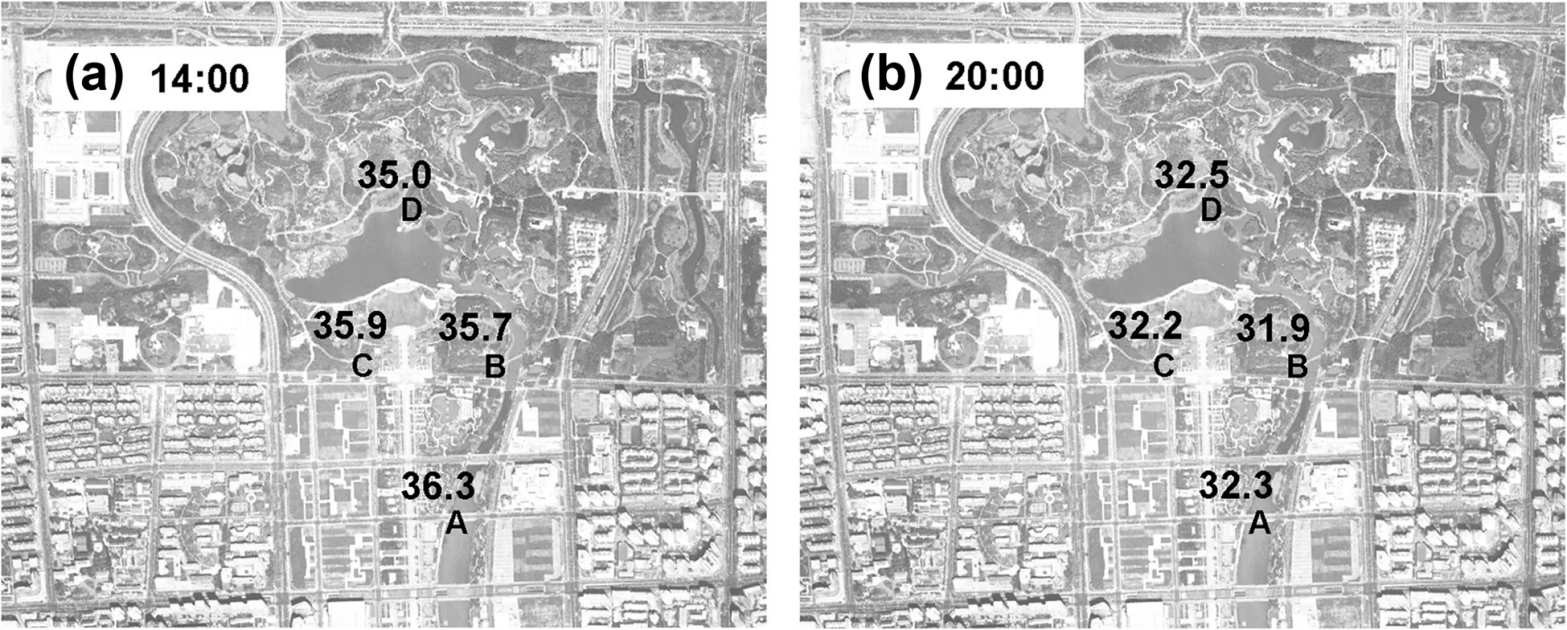
Mean air temperature of different observation sites considering all the field work days (**a**) at 14:00h, (**b**) at 20:00h

To compare the difference of air temperature in four observation sites, we used the average air temperature of four land cover types to represent that of the observation sites namely local temperature effect. Figure 4 shows the result of comparisons among the average air temperatures of four observation sites. At 14:00 h, the average temperature ranged from 35.0 to 36.3 °C at four sites. The hottest place was site A which is located outside the southern entrance of the park, with an average air temperature reaching 36.3 °C. This area has the largest proportion of paved area (44.7 %) and the lowest proportion of trees area (20.1 %). Furthermore, this location is surrounded by some offices and commercial buildings. The continuous heating of the pavement surface by solar radiation and the release of anthropogenic heat contribute to the high air temperature here. The lowest average temperature, 35.0 °C, was obtained at site D, which is located at the center of the park and has the highest proportion of vegetation area (59.4 %). At 20:00 h, the air temperature of site D became the hottest while that of site B became the coldest. These results could be explained by the vegetation character of the corresponding sites. The vegetation in site D is denser and thicker than the vegetation in site B, resulting in a lower sky view factor, which creates better shading efficiency during the day but prevents radiation cooling during the night. In Tel Aviv, a similar result was observed by Potchter et al. [22], indicating that the grass park with few trees tended to be warmer during the day, compared to its surrounding, than 2 other parks with greater tree cover. However, during nighttime the park that contains dense, medium sized trees can create uncomfortable climatic conditions owing to the reduction of wind velocity and increase in relative humidity. This implies that variation in the composition of land cover within a park, such as the amount of tree cover and lawn cover can be expected to affect air temperature. Therefore, we further examined the correlation between air temperature and land cover pattern of each observation site to clarify how land cover influenced local air temperatures in the city of Beijing.

**Fig. 5:**
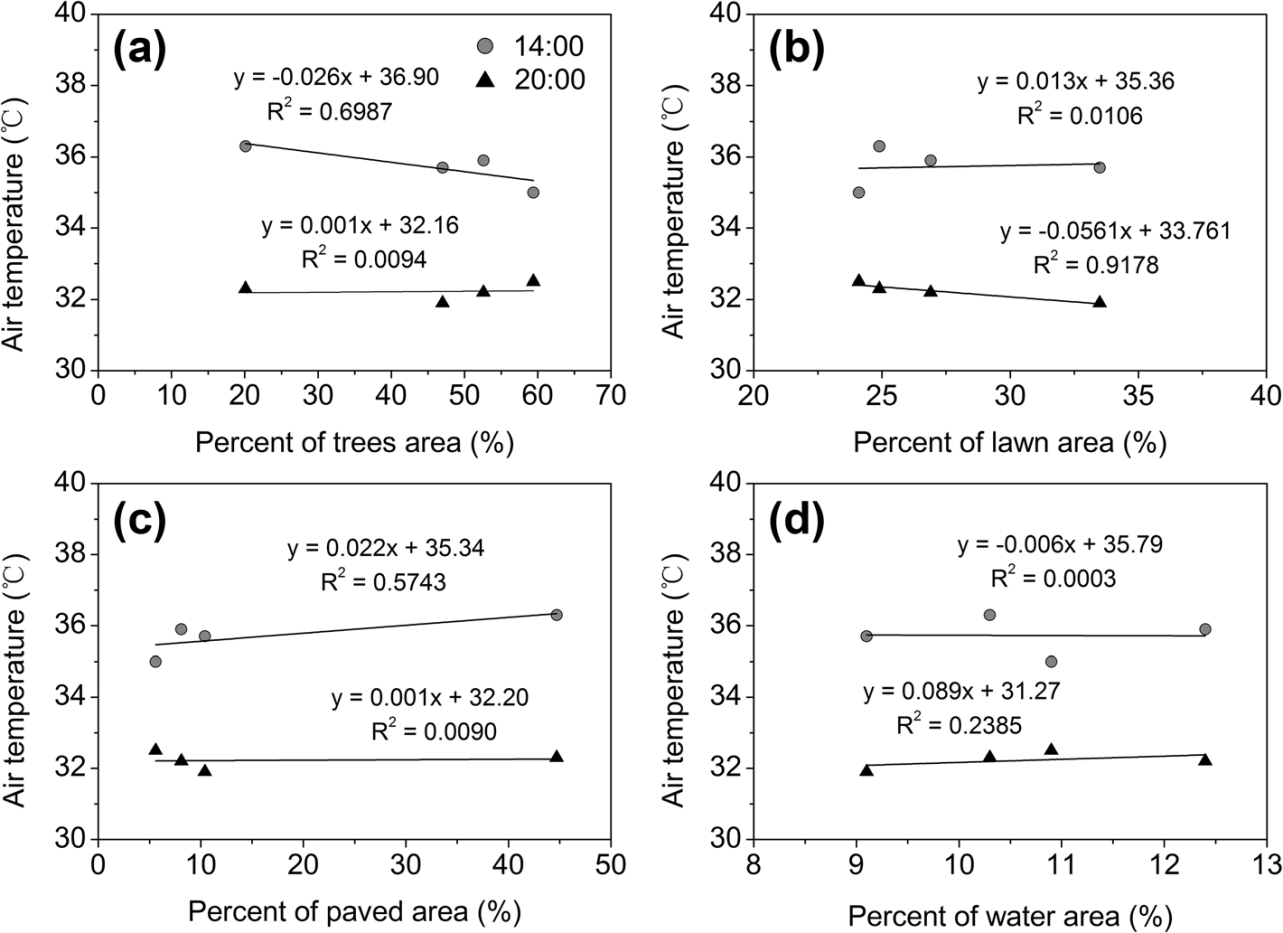
Relationship between air temperature and land cover ratio with regression line (**a**) trees area (%), (**b**) lawn area (%), (**c**) paved area (%), (**d**) water area (%)

Figure 5 shows the correlation between air temperature and the coverage of trees area, lawn area, paved area, and water area within a 125 m in radius of each observation site, represented by the time at 14:00 h and 20:00 h. In spite of the limited size of our field sample, the results pointed out a negative correlation between the air temperature and the percent of tree cover at 14:00 h, as shown in Fig. 5a. About 69.9 % of the variation in air temperature could be explained by the percent tree cover. The air temperature decreased by 0.26 °C as the tree cover ratio increased by 10 %. The result indicated that during daytime, when there was solar radiation, the more the tree coverage, the lower the air temperature is. The vegetation can provide shade and the cooling effect by evapotranspiration; therefore, increasing the tree cover ratio in urban area can mitigate the heat island effect and drop the ambient air temperature. During nighttime, the correlation between air temperature and tree cover was found to be weaker when solar radiation as the main heat source no longer exists. In addition, it shows clearly the change of correlation from negative correlation in the afternoon measurement time to positive correlation in the night measurement time. It means that the tree has an effect on reducing air temperature by shading during hot daytime, however, the tree also reduces the net long-wave loss during nighttime, which in turn, retain and even elevate the air temperature. There was a weak positive correlation between air temperature and lawn cover at 14:00 h, while the lawn cover had the most significant influence on the air temperature at 20:00 h, with a coefficient much higher than others (Fig. 5b). The considerable cooling effect of lawn at night result presumably from its high humidity and exposure to the open sky, which enhances upwelling radiation loss at night. According to the result of this study shown in Fig. 5c, the air temperature always has a positive correlation with paved cover ratio. The air temperature increased by 0.22 °C and 0.01 °C at 14:00 and 20:00 h, respectively, as the paved cover ratio increased by 10 %. At noon in summer, large proportion of paved surfaces absorbs and reflects much incoming radiance, resulting in increased temperature. It also shows that the correlation between air temperature and water cover ratio was pretty poor (Fig. 5d). This result is similar to the one found by Cao et al. [15], which showed that the water body played an unimportant role on PCI formation. This may be due to the fact that the water area was only a very small percentage of the total land cover of each observation site. In general, tree cover and lawn cover are the most main factors which can influence local air temperature during daytime and nighttime, respectively.

## Conclusions

In this paper, we investigated the diurnal variations in air temperature among four types of land cover. The correlations between air temperature and land cover pattern were also discussed. The following conclusions could be drawn:

1. The air temperature difference among four land cover types was more significant during the day than during the night. At noon, the average air temperature differed significantly among four land cover types, which is lower on trees area than on paved area, lawn area, or water area; whereas on night, there was no significant difference among four land cover types.
2. It was also found that the average temperature of each observation site was closely related to the land cover composition. The site with large proportions of paved cover and little vegetation had a very high daytime air temperature, while the site with large proportions of tree cover had a low daytime air temperature. At night, however, an opposite trend was observed.
3. The effect of land cover composition on air temperature demonstrated a diurnal variation and was also dependent on land cover type. We found that site difference in average air temperature at 14:00 h was better predicted by percent tree cover, while average air temperature at 20:00 h was better predicted by percent lawn cover. It was shown that with a 10 % increase in the percent tree cover, the air temperature decreased by 0.26 °C during daytime in summer, by contrast, as the percent lawn cover increased by 10 %, the air temperature decreased by 0.56 °C during nighttime in summer.

This study extended our scientific understanding of the effects of land cover, especial the land cover composition on air temperature in summer. In addition, the findings have important implications for urban green space planning and design. In order to ameliorate the outdoor thermal environment, planners and designers must consider the composition and configuration of the land cover. However, it should be noted that this study was conducted only for one urban green space during a single summer season. Therefore, future work should expand this research to consider more land cover in different metropolitan areas, and expanding the analyses to also include different seasons.
